# Expression of Folliculogenesis-Related Genes in Vitrified Human Ovarian Tissue after Two Weeks *In Vitro* Culture 

**DOI:** 10.22074/cellj.2016.4890

**Published:** 2016-12-21

**Authors:** Zahra Shams Mofarahe, Mojdeh Salehnia, Marefat Ghaffari Novin, Nassim Ghorbanmehr, Mohammad Gholami Fesharaki

**Affiliations:** 1 Department of Biology and Anatomical Sciences, School of Medicine, Shahid Beheshti University of Medical Sciences, Tehran, Iran; 2 Department of Anatomical Sciences, Faculty of Medical Sciences, Tarbiat Modares University, Tehran, Iran; 3 Department of Biotechnology, Faculty of Biological Sciences, Alzahra University, Tehran, Iran; 4 Department of Biostatistics, Faculty of Medicine, Tarbiat Modares University, Tehran, Iran

**Keywords:** Vitrification, Folliculogenesis, Genes Expression, Ovarian Follicles, Human

## Abstract

**Objective:**

This study was designed to evaluate the effects of vitrification and *in vitro* culture of human ovarian tissue on the expression of oocytic and follicular cell-related genes.

**Materials and Methods:**

In this experimental study, ovarian tissue samples were obtained from eight transsexual women. Samples were cut into small fragments and were then assigned to vitrified and non-vitrified groups. In each group, some tissue fragments were divided into un-cultured and cultured (in α-MEM medium for 2 weeks) subgroups. The normality of follicles was assessed by morphological observation under a light microscope using hematoxylin and eosin (H&E) staining. Expression levels of factor in the germ line alpha (*FIGLA*), KIT ligand (*KL*), growth differentiation factor 9 (*GDF-9*) and follicle stimulating hormone receptor (*FSHR*) genes were quantified in both groups by real-time reverse transcriptase polymerase chain reaction (RT-PCR) at the beginning and the end of culture.

**Results:**

The percentage of normal follicles was similar between non-cultured vitrified and non-vitrified groups (P>0.05), however, cultured tissues had significantly fewer normal follicles than non-cultured tissues in both vitrified and non-vitrified groups (P<0.05). In both cultured groups the rate of primary and secondary follicles was significantly higher than non-cultured tissues (P<0.05). The expression of all examined genes was not significantly altered in both non-cultured groups. Whiles, in comparison with cultured tissues non-cultured tissues, the expression of *FIGLA* gene was significantly decreased, *KL* gene was not changed, *GDF-9* and *FSHR* genes was significantly increased (P<0.05).

**Conclusion:**

Human ovarian vitrification following *in vitro* culture has no impairing effects on follicle normality and development and expression of related-genes. However, *in vitro* culture condition has deleterious effects on normality of follicles.

## Introduction

Cryopreservation of ovarian cortical tissue is an alternative strategy for fertility preservation and restoration of ovarian endocrine function (1), especially in premature ovarian failure cases (2) and pre-pubertal girls who need to undergo chemotherapy (3). Vitrification is a novel advanced method for cryopreservation of ovarian tissue by which intracellular fluid is solidified without cellular damage (4). 

The utilization of vitrification solutions containing a mixture of dimethyl sulfoxide (DMSO) and ethylene glycol (EG) has had successful results in preserving the viability of all components of human ovarian cortical tissue including follicular integrity, ovarian stroma and blood vessels (5,8). 

*In vitro* culture of cortical ovarian tissue is not only an efficient method to evaluate the survival and development of follicles after vitrification, but is also ultimately a potential clinical tool for fertility preservation (9). The *in vitro* follicular growth from primordial stages to the development of competent oocytes resulting in live birth has been achieved in a multistep culture system in mice (10). Nevertheless, *in vitro* culture of human follicles is technically challenging due to their larger size, denser ovarian stroma and longer folliculogenesis period. To improve *in vitro* development and survival of follicles, the supplementation of several types of growth factors such as growth differentiation factor 9, leukemia inhibitory factor and activin to the culture media has been recommended (11,12). 

A number of genes are shown to be involved in the normal folliculogenesis process including *FIGLA*, *KL*, *GDF-9* and *FSHR* (13,16). *FIGLA* plays an essential role in the early stages of follicular development. It is expressed in primordial follicles and has a regulatory role in the early events of folliculogenesis (13). In the later stages of follicular development, *GDF-9* is expressed in oocytes of the primary follicle. Its encoded protein is a member of the transforming growth factor β family and plays a principal role in transition of primary follicles to secondary follicles by proliferation and differentiation of granulosa cells (14). 

*KL* is expressed by the granulosa cells in primordial follicles and causes the follicular transition from the primordial to the primary stage (15). *FSHR* is expressed in the granulosa cells of secondary follicles and is involved in follicular transition from the secondary to the antral stage (16). It has been suggested that both the vitrification procedure and *in vitro* culture of ovarian tissue may alter the expression profile of oocytic and follicular cell-related genes (11,17,20). 

There has been a number of studies reporting controversial findings regarding this issue. Isachenko et al. (18). demonstrated that *GAPDH* expression in human vitrified ovarian tissue was dramatically decreased after 16 days of *in vitro* culture. Also, Liebenthron et al. (19) reported a significant increase in *GDF-9* expression at the end of the sixth day of *in vitro* culture of human ovarian tissue. In contrast, Abdollahi et al. (11) reported that vitrification had no effect on the expression of apoptotic-related genes in human ovarian tissue after two weeks of *in vitro* culture. Mazoochi et al. (20) and Fatehi et al. (17) showed that the expression of *p53, Bax, bcl2, Bmp15* and *Gdf-9* had not changed significantly in mouse follicles derived from vitrified ovaries after 12 days of *in vitro* culture. 

To the best of our knowledge, little is known on expression changes in folliculogenesis-related genes after *in vitro* culture of human vitrified ovarian tissue. This study was thus designed to examine potential expression changes of *FIGLA*, *KL*, *GDF-9* and *FSHR* after 14 days of *in vitro* culture of human vitrified ovarian tissue. 

## Materials and Methods

All reagents and materials were obtained from Sigma-Aldrich (Germany) unless mentioned otherwise. 

### Ovarian tissue collection

In this experimental study, ovarian tissue samples were obtained with informed consent from eight transsexual women aged 20-30 years old, undergoing sex reassignment (female to male) surgery by hysterectomy and oophorectomy. The tissues were immediately transferred to the laboratory in pre-warmed and equilibrated Leibovitz’sL-15 medium supplemented with 10 mg/ml human serum albumin (HSA), 100 IU/ml penicillin and 100 μg/ml streptomycin. 

### Preparation of cortical ovarian tissue

The ovarian tissue was transferred to fresh equilibrated Leibovitz’sL-15 medium, and after the medullary parts were removed by a surgical blade, the cortical tissue was cut into small fragments (approximately 2×2×1 mm) under a sterile condition. The fragments taken from each women were then randomly divided into vitrified and non-vitrified groups (n=160 fragments in total). In each non-vitrified and vitrified group, 40 fragments (from 8 women) were considered as non-cultured tissues. Among these tissues, 25 fragments (from 8 women) were fixed in Bouin’s solution for histological evaluation while the rest (from at least 5 women) were stored at -80˚C for subsequent molecular analysis. In each non-vitrified and vitrified group, the remaining fragments (n=40 from 8 women in each group) were cultured *in vitro* for two weeks. Similar to the subgrouping of noncultured tissues, 25 fragments (from 8 women) were fixed in Bouin’s solution for histological evaluation and 15 fragments (from 5 women) were stored at -80˚C for subsequent molecular analysis. 

### Vitrification and warming procedures

The ovarian tissue fragments were vitrified according to the method described by Kagawa et al. (5) with minor modifications. Briefly, the fragments were first washed out in Hanks’ balanced salt solution (HBSS) supplemented with 20% HSA, then equilibrated in HBSS containing 7.5% EG and 7.5% DMSO for 25 minutes. Next, they were transferred into the vitrification solution (20% EG, 20% DMSO and 0.5 M sucrose) for 15 minutes. Finally, the tissue fragments were separately placed in aseptic cryovials containing 100 μl of vitrification solution (pre-placed on nitrogen vapor for 30 seconds), plunged and stored in liquid nitrogen for a week. 

The fragments were warmed by plunging the vials in a 37˚C water bath until complete melting of the samples. The samples were then transferred quic*KL*y into 1 M sucrose in HBSS for 3 minutes at 37˚C and moved into 0.5 M sucrose for 5 minutes at room temperature. Finally, the fragments were equilibrated in α-MEM medium. 

### In vitro culture of cortical ovarian tissue

Vitrified and non-vitrified tissue fragments (80 fragments in total) were cultured individually in 24-well culture plates with inserts (Millicell Culture Plate Inserts, France) for 2 weeks in 400 μl of α-MEM supplemented with sodium bicarbonate (2.2 mg/ml), sodium pyruvate (25 μg/ml), HSA (5 mg/ml), penicillin G (0.1 mg/ml), streptomycin (0.1 mg/ml), insulin, transferring selenium (ITS), human recombinant follicle stimulating hormone (0.5 IU/ml) and ascorbic acid (50 μg/ml) at 5 % CO_2_ and 37˚C. Half of the culture medium within each well was removed and replaced with fresh medium every other day. After 2 weeks, cultured tissues in non-vitrified and vitrified groups were fixed in Bouin’s solution for histological evaluation (n=25 in each group) and the rest were stored at -80˚C for subsequent molecular analyses.

### Histological evaluation by hematoxylin and eosin

A total of 100 fragments from all four sub-groups were fixed in Bouin’s solution for 20 hours at room temperature. They were subsequently processed and embedded in paraffin wax, and serially sectioned at 5 μm thickness. Each tenth section of every fragment was mounted on a glass slide and stained with hematoxylin and eosin (H&E). The slide was then examined for follicular counting, field by field under the ×10 objective of the light microscope. In order to avoid recounting the follicles, follicles with an obvious nucleus of oocytes were counted only. The follicles were classified as primordial, primary and secondary according to a previous classification (21). Primordial follicles had one layer of flattened follicular cells, primary follicles had one layer of cuboidal follicular cells and secondary follicles had two or more layers of cuboidal granulosa cells. Atretic follicles had pyknotic oocyte nuclei, shrunken ooplasms or disorganized follicular cells. 

### RNA extraction and cDNA synthesis

Total RNA was extracted from 60 non-cultured and cultured fragments in both vitrified and nonvitrified groups using TRIzol (Invitrogen, USA) according to the manufacturer’s instructions. RNA samples were treated with DNase to remove any genomic DNA contamination prior to cDNA synthesis. The RNA concentration was determined by spectrophotometry and adjusted to a concentration of 250 ng/µl. Finally, cDNA was synthesized from 1000 ng of the extracted RNA by using a commercial kit (Thermo Scientific, EU) according to the manufacturer’s instructions at 42˚C for 60 minutes. The synthesized cDNA samples were stored at -20˚C until utilized. 

### Real-time reverse transcriptase polymerase chain reaction

The primers for real-time RT-PCR were designed (Table 1) and primer 3 (http://sourceforge.net/ projects/primer3/) and synthesized by Generay Biotech Co. (China). in the same run. One microliter of cDNA, 1 µl of the mixture of forward and reverse primers One-step RT-PCR was performed on the Applied Biosystems (UK) real-time thermal cycler according to QuantiTect SYBR Green RT-PCR Kit (Applied Biosystems, UK). The housekeeping gene, *β-ACTIN*, was used as an internal control. For each sample, the reference gene and the target genes were amplified and 10 µl of SYBR Green Master Mix were used per 20 µl of reaction volume. Negative samples were no-template controls. After completing the PCR run, melt curve analysis was used to confirm the amplified product and record the Ct values. When a melting curve peak was not observed, the cDNA of another fragment would be substituted. Real-time heating conditions included a holding step at 95˚C for 5 minutes cycles of 95˚C for 15 seconds, 58˚C for 30 seconds and 72˚C for 15 seconds, followed by a melt curve step at 95˚C for 15 seconds, 60˚C for 1 minute and 95˚C for 15 seconds. The relative quantification of target genes was determined using the Pfaffl method (22). The real-time RT-PCR experiments were done in triplicate for each sample. 

### Statistical analysis

Statistical analysis was undertaken using SPSS (IBM SPSS statistics 22). Variables were displayed as mean ± SE and percentage. The results of follicular counting and real-time RT-PCR data were compared by paired-sample t-test and bootstrapping. P values less than 0.05 were considered as statistically significant. 

### Ethical considerations

The present study was approved by the Ethics Committee of the Shahid Beheshti University of Medical Sciences (no.172). Informed consent was obtained from all participants. 

**Table 1:** The details of primers used for real-time reverse transcriptase polymerase chain reaction (RT-PCR) assays


Accession number	Target gene	Primer sequence (5ˊ-3ˊ)	Product size (bp)

NM_001101.3	*β-ACTIN*	F: TCAGAGCAAGAGAGGCATCC	187
		R: GGTCATCTTCTCACGGTTGG	
NM_001004311.3	*FIGLA*	F: TCGTCCACTGAAAACCTCCAG	76
		R: TTCTTATCCGCTCACGCTCC	
NM_000899.4	*KIT LIGAND*	F: AATCCTCTCGTCAAAACTGAAGG	163
		R: CCATCTCGCTTATCCAACACTGA	
NM_005260	*GDF-9*	F: TCCACCCACACACCTGAAAT	147
		R: GCAGCAAAACCAAAGGAGGA	
NM_181446.2	*FSHR*	F: CTGGCAGAAGAGAATGAGTCC	157
		R: TGAGGATGTTGTACCCGATGATA	


### Results

#### Histological observation of ovarian tissue 

**Fig.1:**
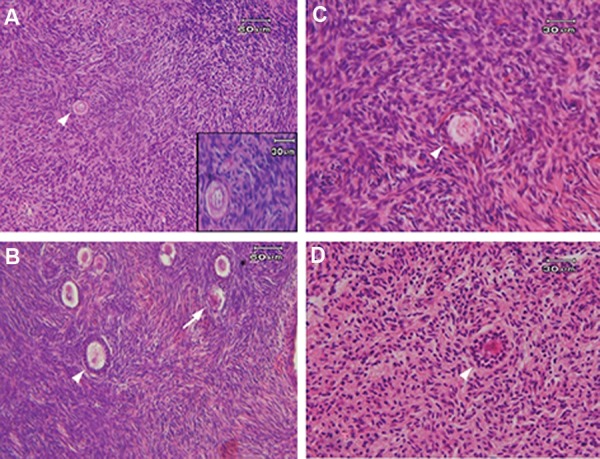
Hematoxylin and eosin (H&E) staining of non-cultured and cultured human ovarian cortical sections in both vitrified and nonvitrified groups. A. The morphology of primordial follicle (white arrow heads) was observed in non-cultured non-vitrified tissue, B. The normal morphology of primary follicle (white arrow heads) and atretic follicle (white arrow) were seen in cultured nonvitrified tissue, C. The morphology of primary follicle (white arrow heads) was observed in non-cultured vitrified tissue, and D. The normal morphology of secondary follicle (white arrow heads) was seen in cultured vitrified tissue.

The morphology of non-cultured and cultured ovarian cortical sections in both vitrified and non-vitrified groups is shown in Figure 1. The morphology of follicles and stromal cells in vitrified tissues was similar to those of the nonvitrified group. Normal follicles contained spherical oocytes, surrounded by granulosa cells which were in close contact with each other, and stromal cells which had spindle-shaped nucleus and a delicately diffused chromatin. In contrast, atretic follicles had pyknotic oocyte nuclei, shrunken ooplasms or disorganized follicular cells. At the end of culture period, there were growing follicles (primary and secondary) in vitrified and non-vitrified tissues. 

#### Percentage of normal follicles in non-cultured ovarian tissues

A total of 500 follicles were counted in 50 noncultured ovarian fragments in the non-vitrified and vitrified groups. The rate of morphologically normal follicles at different developmental stages in both groups is shown in Table 2. The proportion of normal follicles was 91.3% in non-vitrified and 87.4% in vitrified tissues. Among the normal follicles, there was no significant difference in the proportion of primordial, primary and secondary follicles between the two groups (P>0.05). 

**Table 2:** The rate of normal follicles at different developmental stages before and after *in vitro* culture of human ovarian tissues


Groups	No. of total follicles	No. of normal follicles(Mean% ± SE)	No. of primordial follicles(Mean% ± SE)	No. of primary follicles(Mean% ± SE)	No. of secondary follicles(Mean% ± SE)

Non-cultured vitrified	230	201/230^*^^*^(87.4 ± 1.5)	135/201^*^^*^(58.7 ± 2.8)	57/201^*^^*^(24.8 ± 2.9)	9/201^*^^*^(3.9 ± 0.4)
Non-cultured non-vitrified	270	247/270^*^(91.3 ± 2.1)	166/247^*^(61.4 ± 5.1)	68/247^*^(25.1 ± 4.9)	13/247^*^(4.8 ± 1.8)
Cultured vitrified	277	205/277^*^^*^(74 ± 3.8)	59/205^*^^*^(21.5 ± 3.5)	120/205^*^^*^(43.2 ± 3.8)	26/205^*^^*^(9.3 ± 2.3)
Cultured non-vitrified	299	249/299^*^(83.2 ± 1.6)	60/249^*^(20 ± 4.4)	151/249^*^(50.4± 2.7)	38/249^*^(12.8 ± 3.9)


There was no significant difference between the vitrified and non-vitrified groups with and without culture in all columns (P>0.05). ** ; Significant differences between non-cultured non-vitrified and cultured non-vitrified sub-groups in the same column (P<0.05) and * ; Significant differences between non-cultured vitrified and cultured vitrified sub-groups in the same column (P<0.05).

#### Percentage of normal follicles in cultured ovarian tissues

A total of 576 follicles were counted and analyzed in 50 cultured ovarian cortical fragments in the non-vitrified and vitrified groups. The rate of morphologically normal follicles at different developmental stages is given in Table 2. The proportion of normal follicles was 83.2% in non- vitrified and 74% in vitrified tissues. Among the normal follicles, there was no significant difference in the proportion of primordial, primary and secondary follicles between the two groups (P>0.05).

#### Comparison of normal follicle rate in non-cultured and cultured ovarian tissues

In both cultured tissues, in comparison with non-cultured tissues, the rate of normal follicles was significantly lower while the rate of atretic follicles was significantly higher (P<0.05). The proportion of primordial follicles was significantly lower, however, the proportion of primary and secondary follicles was higher in both cultured tissues compared with their respective non-cultured groups (P<0.05).

#### Expression of folliculogenesis-related genes in non-cultured ovarian tissues

**Fig.2:**
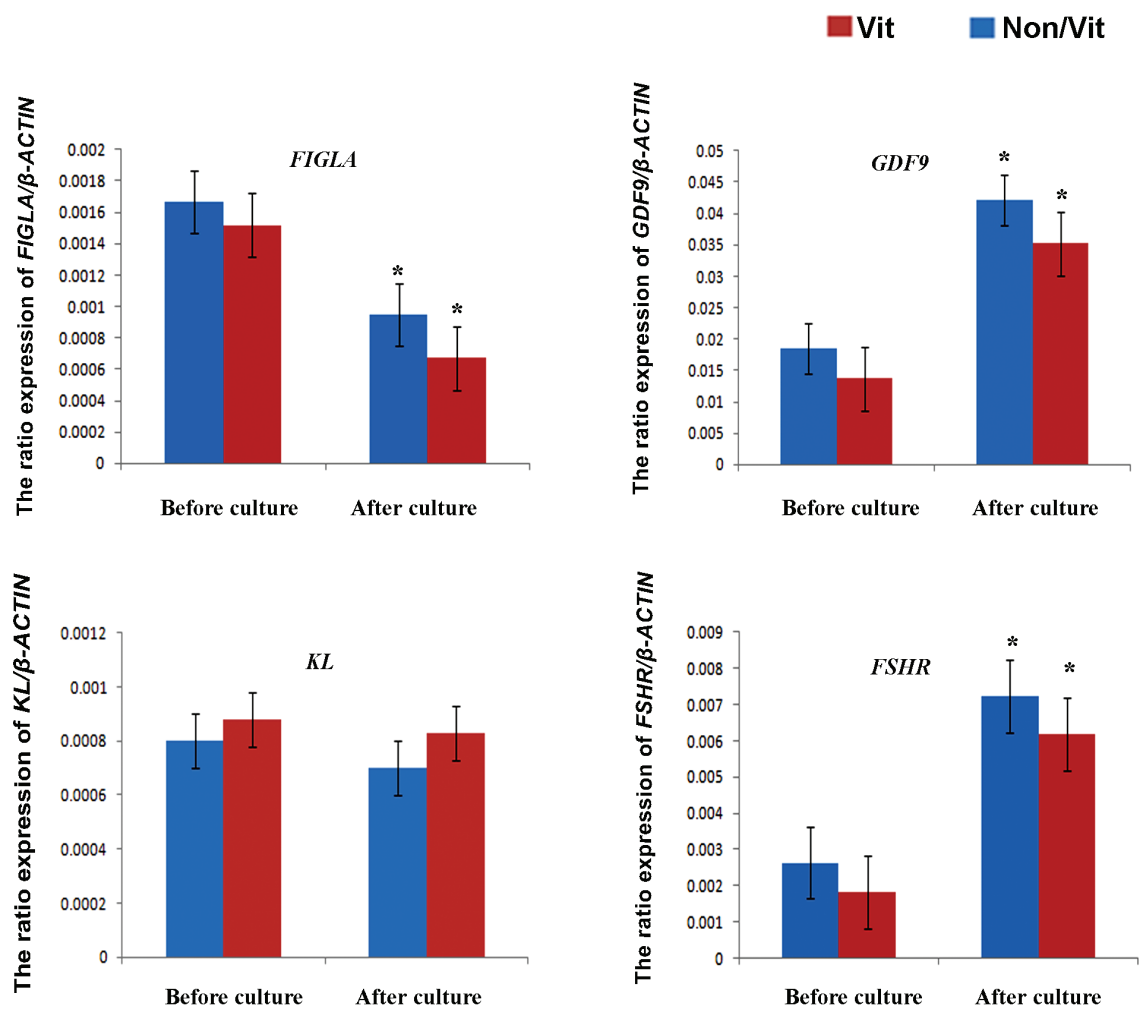
Expression of folliculogenesis-related genes in non-cultured and cultured human ovarian cortical tissues of vitrified and non-vitrified groups. Values are mean ± SE. There was a significant increase in the expression of *GDF-9* and *FSHR* along with a decrease in *FIGLA* expression in cultured tissues when compared with non-cultured samples (*).

The relative expression of *FIGLA*, *GDF-9*, *KL* and *FSHR* to *β-ACTIN* in the non-vitrified group before culture was 16.7×10^-4^, 18.6×10^-4^ , 8×10^-4^and 26.4×10^-4^. In the non-cultured-vitrified group, these were 15.2×10^-4^, 13.7×10^-4^, 8.8×10^-4^ and 18.3×10^-4^ respectively (Fig.2). There was no significant difference in the expression of all examined genes between the two non-cultured tissues (P>0.05).

#### Expression of folliculogenesis-related genes in cultured ovarian tissues

The relative expression of *FIGLA*, *GDF-9*, *KL* and *FSHR* to *β-ACTIN* in the non-vitrified group after culture was 9.5×10^-4^ , 421.8×10^-4^, 7×10^-4^and 72.4×10^-4^while in the vitrified group, it was 6.7×10^-4^, 312.1×10^-4^, 8.3×10^-4^and 61.8×10^-4^ respectively (Fig.2). There was no significant difference in the expression of all examined genes between cultured-vitrified and cultured- non-vitrified sub-groups (P>0.05).

### Comparison of folliculogenesis-related genes expression in non-cultured and cultured ovarian tissues 

In both cultured tissues in comparison to noncultured tissues, *FIGLA* gene expression was significantly decreased, *KL* gene expression was not changed, the expression of *GDF-9* and *FSHR* genes was significantly increased (P<0.05). 

### Discussion

Histological analysis revealed that the rate of morphologically normal follicles at different developmental stages was not only similar in the non-vitrified and vitrified ovarian tissues before culture, but was also similar after *in vitro* culture. This suggests that this procedure of vitrification had no impairing effect on human ovarian tissue morphology after warming and two weeks of culture. The competence of this method may be due to the use of EG, DMSO and sucrose as cryoprotectants. EG has a rapid cell diffusion and also highly compatible with other cryoprotectants. Adding sucrose in the vitrification solution may decrease the risk of intracellular water crystallization thus reducing the cryo damage to the tissue (23). This observation is similar to those in other species (5,24). 

We also observed that the proportion of growing follicles (primary and secondary follicles) was significantly increased in parallel with a decrease in the rate of primordial follicles in both cultured groups. Therefore, *in vitro* culture of human ovarian tissue is a reasonable method to stimulate the growth of follicles and evaluate the viability of ovarian follicles in the vitrified tissues. Similar results to these were also published by others (11,12,24). However, in spite of a high rate of growing follicles in both vitrified and nonvitrified cultured groups, there were more atretic follicles in cultured tissues than non-cultured samples. It seems that the higher incidence of atretic follicles in cultured tissues may be due to the insufficient culture medium used in this study for normal follicular development and thus adding some growth factors is recommended. It has been suggested that *in vitro* culture conditions have more deleterious effects on the rate of normal follicles than the vitrification procedure. Consistent with our results, a number of studies have reported that the percentage of normal follicles decreases significantly in human vitrified and non-vitrified ovarian tissues after culture in basic medium. Addition of growth factors such as growth differentiation factor 9, leukemia inhibitory factor and activin in basic medium is thought to increase the number of normal follicles in human ovarian tissue at the end of the culture period (11,12,25,26). 

Lack of differential expression of *FIGLA*, *KL*, *GDF-9* and *FSHR* between the non-vitrified and vitrified ovarian tissue samples provide evidence that vitrification by DMSO, EG and sucrose has no impairing effects on the expression of developmental genes. On the other hand, expression of these genes is essential for transition of follicles to the next stage of development (17). Similar observations to ours were reported by Abir et al. (26) where anti-apoptotic genes were similarly expressed between non-vitrified and vitrified human ovarian tissue after 14 days of culture. Also, the vitrification of mice ovarian tissue using EG, DMSO and sucrose showed similar conditions to fresh tissue based on the expression of *Gdf-9* and *Bmp15* after 12 days of *in vitro* culture (17). In contrast, Isachenko et al/ demonstrated that after vitrification of human ovarian tissue, *GAPDH* expression was decreased (18). One suggestion for these inconsistent results may be due to the vitrification procedure and the type of cryoprotectants used in different studies. 

Our results indicated that a significant increase in the *GDF-9* and *FSHR* genes expression along with a decrease in *FIGLA* gene expression in cultured tissues in comparison with non-cultured samples (P<0.05). Given that *FIGLA* is expressed in primordial follicles, and *GDF-9* and *FSHR* are expressed in growing follicles, our expression results revealed the transition of follicles from the primordial to the growing stage (14,16). Therefore, differential expression of these genes may be used as biomarkers of follicular development. 

## Conclusion

Human ovarian vitrification following *in vitro* culture has no impairing effects on follicular normality and development, and expression of related genes. 
